# Building‐Tailored Hierarchical Electrochromism with Optimized Control Strategies

**DOI:** 10.1002/advs.202513530

**Published:** 2025-10-16

**Authors:** Shuangdui Wu, Jiawei Sun, Zhoujie Duan, Xiaolei Li, Jun Xiao, Hongli Sun, Borong Lin

**Affiliations:** ^1^ Key Laboratory of Eco Planning & Green Building Ministry of Education Tsinghua University Beijing 100084 China; ^2^ Department of Building Science Tsinghua University Beijing 100084 China; ^3^ College of Architecture and Environment Sichuan University Chengdu 610065 China; ^4^ Interdisciplinary Materials Research Center, Institute for Advanced Study Chengdu University China; ^5^ State Key Laboratory of Hydraulics and Mountain River Engineering Sichuan University Chengdu 610065 China

**Keywords:** building‐centric paradigm, control strategy, electrochromic smart window, multi‐band modulation

## Abstract

Electrochromic smart windows struggle with a material‐application disconnect. So, a building‐centric paradigm is established to bridge this gap through dual breakthroughs: multi‐band hierarchical material and control strategy. An electrochromic device (ECD) based on Prussian blue (PB) with multi‐band hierarchical modulation is demonstrated, integrating Fe_4_[Fe(CN)_6_]_3_ and Nb_18_W_16_O_93_ film electrodes, where hierarchical regulation relies on potassium ion shuttling (rocking‐chair mechanism) and cation‐anion co‐intercalation dynamics. It enables four spectral customization states: transparent heating (S1), zero‐energy bright heating (S2), daylight‐preserving bright cooling (S3), and maximum‐blocking dark cooling (S4). An Electrochromic window (ECW) constructed with such ECD, when coupled with optional intelligent control strategies, transforms these capabilities into unprecedented energy savings. The load‐responsive strategy focuses on building internal demands, leverages the zero‐energy S2 state for >75% of summer operation, achieving 25.4% cooling energy reduction versus normal windows. The climate‐adaptive strategy, centered on external environmental constraints and guided by “location‐specific adjustment”, reveals >50% performance disparities between control parameters, enabling location‐optimized operation such as 56.9% summer energy savings even in cold Helsinki. By embedding tunable spectral control into the building energy management logic, material, and control strategy innovations have jointly paved a scalable path from laboratory innovation to building application.

## Graphical abstract

This study addresses the material‐application disconnect in electrochromic smart windows by establishing a building‐centric paradigm. It develops a Prussian blue (PB)‐based electrochromic device (ECD) with four spectral states via dual mechanisms, and proposes two control strategies. The load‐responsive strategy cuts summer cooling energy by 25.4%, while the climate‐adaptive one achieves 56.9% savings in Helsinki's summer, bridging lab innovation to building application.

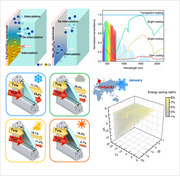

## 1 Introduction

The building sector accounts for 39% of global primary energy consumption, exceeding the industrial (33%) and transportation (28%) sectors—a statistic that underscores the pivotal role of building energy use in global energy demand.^[^ ^1^, ^2^ ^]^ Traditional windows, as passive components, lack the ability to dynamically regulate solar radiation, failing to adjust the transmittance of visible (VIS) or near‐infrared (NIR) light. This limitation leads to the continuous operation of lighting and air‐conditioning system, consuming substantial energy to maintain indoor comfort.^[^ ^3^ ^]^ Electrochromic technology stands out for its ability to dynamically adjust light transmittance, enabling active control of indoor lighting and temperature in innovative energy‐efficient applications.^[^ ^4^, ^5^, ^6^, ^7^, ^8^, ^9^ ^]^ The functional layer of electrochromic windows (ECWs) includes electrochromic layer, electrolyte layer and ion storage layer, the electrochromic performance of which is dominated by electrochromic layer. Traditional electrochromic smart windows are mostly achieved through the complementarity of tungsten oxide (WO_3_) and nickel oxide (NiO). The WO_3_, a typical cathode coloration electrochromic material, shows a transparent state with high solar irradiance transmittances and a deep blue colored state.^[^ ^10^ ^]^ Under reduction potential, crystalline WO_3_ typically exhibits significant optical modulation in the NIR region, while amorphous WO_3_ demonstrates synergistic regulation of VIS and NIR light.^[^ ^11^, ^12^, ^13^ ^]^ For achieve the tunable near‐infrared and visible‐light transmittance,^.[^ ^14^, ^15^ ^]^ cathode coloration electrochromic oxides (WO_3_, TiO_2_, Nb_2_O_5_, etc.) with special nanostructures exhibit dual‐band modulation have been study in recent, at moderate reduction potentials, they regulate only near‐infrared light, whereas at lower reduction potentials, they co‐regulate VIS and NIR light.^[^ ^16^, ^17^, ^18^, ^19^, ^20^, ^21^, ^22^, ^23^ ^]^

Current research on smart windows primarily focuses on material development and verification of energy‐saving effects in specific scenarios.^[^ ^24^ ^]^ However, it faces a critical bottleneck of “product availability without effective utilization” due to inadequate scenario adaptability.^[^ ^25^, ^26^, ^27^ ^]^ On one hand, the excessive pursuit of extreme performance parameters in materials ignores the design of application‐specific control strategies, causing significant degradation of laboratory‐proven high performance during engineering implementation.^[^ ^28^, ^29^, ^30^ ^]^ On the other hand, product application studies remain at the technical validation stage, failing to reverse‐feed evaluation results into material and device development, thus disconnecting demand from technological iteration. In fact, there is no one‐size‐fits‐all material solution. This study emphasizes a demand‐oriented approach, where precise matching between material performance and scenario‐specific requirements is achieved through dynamic control strategies to maximize the engineering value of smart window technologies.

Against this backdrop, this study introduces a building‐centric paradigm for electrochromic technology development. We analyzed the broad application domains of electrochromic devices (ECDs) by evaluating two traditional electrochromic materials. Our analysis leads to the conclusion that Fe_4_[Fe(CN)_6_]_3_ (Prussian Blue, PB)‐based ECDs are well‐suited for versatile applications.^[^ ^31^, ^32^, ^33^, ^34^ ^]^ Notably, we demonstrate a multi‐band hierarchically controlled electrochromic device (ECD), whose modulation performance relies on the cationic rocking‐chair and cation‐anion co‐intercalation mechanisms.^[^ ^35^, ^36^ ^]^ This ECD is assembled using Fe_4_[Fe(CN)_6_]_3_ and Nb_18_W_16_O_93_ film electrodes. The switching of transparent heating to bright heating state is dominated by the reciprocating movement of potassium ions (rocking‐chair),^.[^ ^36^ ^]^ while the transition of bright cooling to dark cooling arises from cation‐anion co‐intercalation. Enabled by tunable spectral and charge balance design, the electrochromic window (ECW) constructed with such ECD allows extensive color and spectral modulation while maintaining excellent hierarchical and multi‐band functionality. Furthermore, we establish two dynamic control strategies directly addressing the material‐application disconnect: 1) a hierarchical energy‐saving control strategy based on building load demand, and 2) a climate‐adaptive strategy tailored to outdoor temperature characteristics. These strategies go beyond mere static comparisons: by integrating the cationic rocking‐chair and cation‐anion co‐intercalation mechanisms into the operational logic of building energy systems, they achieve precise matching between spectral modulation and spatiotemporal energy demand scenarios.

## 2 Results and Discussion

### 2.1 Applicability Analysis of Electrochromic Material Smart Windows

**Figure 1:**
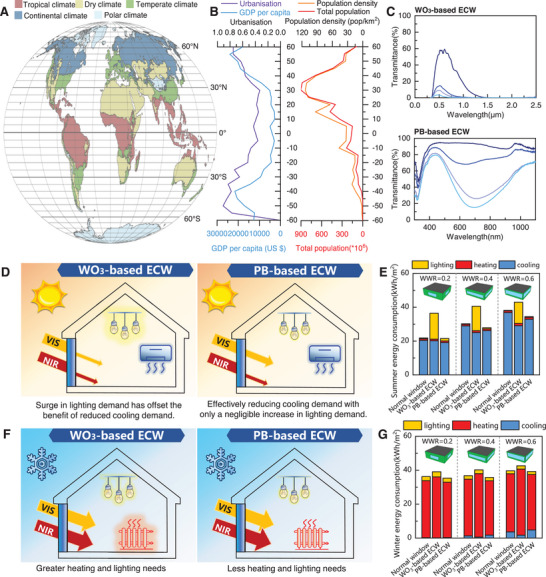
Global population distribution and urbanization analysis for the wide applicability of electrochromic smart windows (ECWs). A) Köppen climate zones. B) Latitudinal distribution of population density and economic development. C) Typical transmittance spectra of ECW based on WO_3_ and PB. D) Summer energy demand comparison diagram of ECW based on WO_3_ and PB. The thickness or color rendering intensity of the glow indicates the magnitude of the corresponding sub‐item energy consumption. E) Quantification of summer energy savings of WO_3_ and PB‐Based ECWs in typical Beijing office buildings via EnergyPlus simulations. F) Winter energy demand comparison diagram of ECW based on WO_3_ and PB. G) Quantification of winter energy savings of WO_3_ and PB‐Based ECWs in typical Beijing office buildings via EnergyPlus simulations.

As depicted in **Figure** **1**A, Köppen climate zones exhibit pronounced latitudinal zonation.^[^ ^37^ ^]^ The global population is predominantly concentrated in the Northern Hemisphere, particularly within the 20°N–40°N latitude band, with population density decreasing sharply as climate conditions become more extreme. Economic indicators, such as GDP and urbanization rates, display a “mirror‐symmetric” relationship with latitude: values are lowest near the equator (15°N‐15°S) and increase gradually toward the mid‐latitudes.

Thus, mid‐latitude temperate climate zones serve as the epicenter of global population aggregation and economic activities (Figure 1B).^[^ ^38^ ^]^ The region's distinct four‐season climate presents dual challenges of winter heating and summer cooling, necessitating smart window designs with dynamic light‐heat regulation capabilities. Specifically, intelligent fenestration systems must exhibit hierarchical spectral control characteristics to effectively distinguish and address seasonal thermal management demands—optimizing winter solar gain while mitigating summer overheating, thereby realizing the adaptive application potential of ECWs.

Building energy simulations were conducted for representative building types in Beijing, a typical mid‐latitude city, considering various window‐to‐wall ratios (WWRs). Conventional ECDs predominantly utilize WO_3_ as the core material, exhibiting the characteristic optical spectrum depicted in Figure 1C. The pronounced absorption of WO_3_ in its reduced (colored) state within the VIS spectrum (600–800 nm) significantly reduces cooling energy consumption during summer.^[^ ^39^ ^]^ However, this inherent limitation in visible light transmittance (T_vis_) concurrently necessitates increased artificial lighting energy usage (Figure 1D,E).

In contrast, PB exhibits a characteristic absorption peak in the wavelength of 420–980 nm by K^+^ intercalation (Figure 1C), precisely at the boundary between VIS and NIR light regions.^[^ ^40^ ^]^ In its colored state, PB maintains high transmittance (>60%) in the 500–600 nm VIS band while suppressing NIR thermal radiation (e.g., only 6.4% transmittance in the deep‐blue state), enabling a balance between natural daylighting and thermal load management. In the transparent state, its high optical transmittance enhances natural lighting and solar heating, making PB‐based ECW ideal for balancing cooling, heating, and lighting energy demands in mixed‐climate regions across seasons. This dual‐mode spectral tunability underscores their potential for large‐scale adoption in energy‐efficient architecture.

As shown in Figure 1E,G, PB‐based ECW consistently exhibit superior energy‐saving efficiency compared to normal window and conventional WO_3_‐based ECW and the advantage becomes increasingly significant as the WWRs increases. When the WWRs increases from 0.2 to 0.6, ECW achieves a heating energy‐saving rate increase from 2.8% to 4.6% in winter and a cooling energy‐saving rate increase from 6.4% to 10.7% in summer, with its summer lighting energy consumption remaining nearly unchanged compared to ordinary windows while traditional WO_3_ shows a sharp increase. By applying an appropriate voltage, the colored state of PB‐based ECW reduces NIR transmittance (T_nir_), thereby minimizing solar heat gain and air‐conditioning loads. Simultaneously, these devices maintain high optical transmittance in the visible spectrum, especially in the visually sensitive range, ensuring effective natural lighting and preventing increased lighting energy consumption. Therefore, the spectral characteristics of PB‐based ECW demonstrate strong adaptability and invaluable application potential for various building types across broad mid‐latitude regions.

### 2.2 Hierarchically Regulated Electrochromic Device (ECD) Assembled with Fe_4_[Fe(CN)_6_]_3_ and Nb_18_W_16_O_93_ Film Electrodes

**Figure 2:**
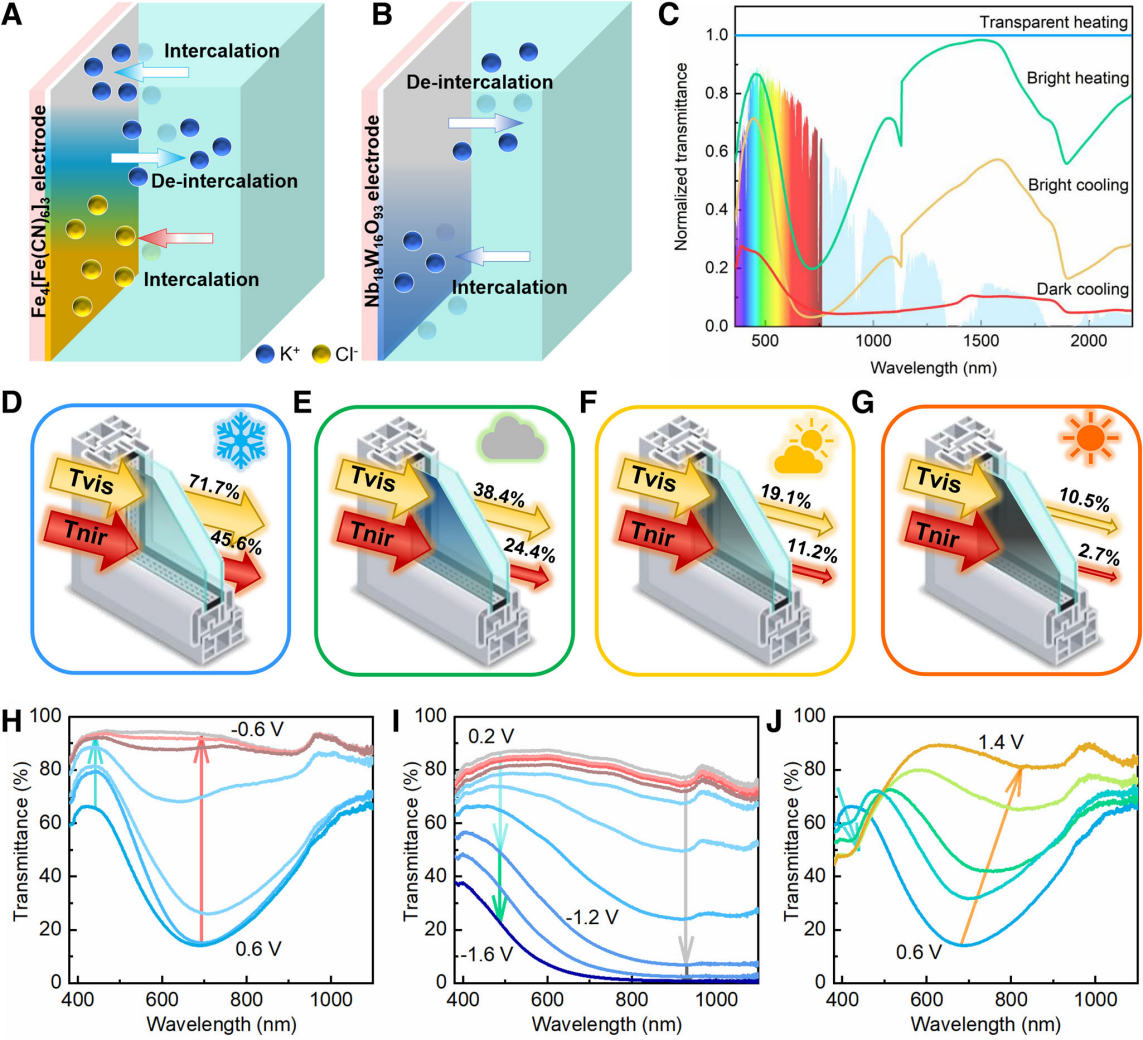
Properties and mechanism of a multi‐band hierarchically controlled electrochromic device (ECD) assembled with Fe_4_[Fe(CN)_6_]_3_ and Nb_18_W_16_O_93_ film electrodes via rocking‐chair and cation‐anion co‐intercalation. A) Schematic diagram of color switching in Fe_4_[Fe(CN)_6_]_3_ film electrode during K⁺ and Cl^−^ ions intercalation/deintercalation processes. B) Schematic diagram of color switching in Nb_18_W_16_O_93_ film electrode during K⁺ ion intercalation/deintercalation. C) Normalized transmittance spectra of the ECD in four states at the wavelength range of 380–2200 nm (Figures S1 and S2, Supporting Information). D) Applicable scenarios for transparent heating condition. E) Applicable scenarios for bright heating condition. F) Applicable scenarios for bright cooling condition. G) Applicable scenarios for dark cooling condition. H) In‐situ transmittance spectrum of Fe_4_[Fe(CN)_6_]_3_ film electrode as a function of bias during the K^+^ intercalation (Figure S3, Supporting Information). I) In‐situ transmittance spectrum of Nb_18_W_16_O_93_ film electrode as a function of bias during the K^+^ intercalation (Figure S4, Supporting Information). J) In‐situ transmittance spectrum of Fe_4_[Fe(CN)_6_]_3_ film electrode as a function of bias during the Cl^−^ intercalation (Figure S5, Supporting Information). Transparent heating, bright heating, bright cooling and dark cooling states are defined as S1, S2, S3, and S4, respectively.

It is well established that the blue‐transparent switching in Fe_4_[Fe(CN)_6_]_3_ is attributed to cationic intercalation.^[^ ^31^, ^41^, ^42^ ^]^ However, the less‐known mechanism of blue‐green or blue‐yellow switching via anionic (Cl^−^) intercalation remains understudied (**Figure** **2**A). Therefore, in order to enhance the functionality of PB‐based devices, a wider operating bias and a greater variety of intercalated ions should be used in PB‐based ECDs. Thus, the Nb_18_W_16_O_93_ is employed as complementary electrode for cations storage. It can provide a higher operating bias than tungsten oxide.^[^ ^43^, ^44^, ^45^, ^46^, ^47^ ^]^ Due to the nearly identical ionic conductivities (73.5 and 76.4 S cm^−2^ mol^−1^ for K^+^ and Cl^−^, respectively) and mobilities (7.6 × 10^−4^ and 7.9 × 10^−4^ cm^2^ V^−1^ s^−1^), K^+^ and Cl^−^ ions were chosen as electrolyte ions to enable fast and complementary kinetic responses. PAM (polyacrylamide) hydrogel containing KCl is used as the electrolyte. PAM hydrogels not only exhibit high transparency, high ionic conductivity, and strong interfacial adhesion similar to aqueous solutions but also prevent the dissolution of PB during redox reactions, which is a common issue in aqueous electrolytes.^[^ ^47^, ^48^, ^49^, ^50^, ^51^ ^]^ More importantly, the reasonable charge storage capacity of Nb_18_W_16_O_93_ is complementary to PB in a matched potential window, so that the full device with large optical modulation, fast response and long‐term durability. These are considered based on reasons such as intelligence, fast switching, and low‐price.

To clearly illustrate the transmittance variation trend under different biases, the original transmittance curves were normalized. As shown in Figure 2B, the ECD assembled with Fe_4_[Fe(CN)_6_]_3_ (PB) and Nb_18_W_16_O_93_ film electrodes can achieve at least four coloration states—transparent heating (S1), bright heating (S2), bright cooling(S3), and dark cooling (S4)—for applications in diverse climatic conditions (Figure S1, Supporting Information). By applying a bias of 1.5 V, Fe_4_[Fe(CN)_6_]_3_ undergoes a transformation to Prussian white (PW, transparent) via K^+^ ion (1.33 Å) intercalation. Meanwhile, Cl^−^ ions (1.81 Å) accumulate on the Nb_18_W_16_O_93_ electrode rather than diffusing into its lattice, attributed to their larger ionic radius. In this state, the ECD exhibits transparency with high transmittance in both visible and near‐infrared light (Figure 2C, blue line, S1). Once the bias is switched back to open circuit potential (OCP), the ECD reverts to its initial state (bright heating, green line, S2) as Prussian white (PW) transforms back to PB via K^+^ deintercalation. In bright heating state, the OCP of ECD is near zero. Due to the combination of the transparent Nb_18_W_16_O_93_ film electrode and the intrinsic blue color of the PB film electrode, the as‐assembled ECD exhibits a blue appearance. This exemplifies the primary cationic behavior in rocking‐chair type electrochromic devices.

Compared to the transparent state (Figure 2D), the bright heating state can block solar irradiation, with most of the blocked energy derived from VIS light (460–780 nm) and a small portion from NIR light (780–1600 nm). This results in minimal infrared heating (Figure 2E). When a negative bias of −1.8 V is applied, the Nb_18_W_16_O_93_ film turns blue via K^+^ intercalation, while PB transforms into Prussian green (PG) through Cl^−^ intercalation. This stage represents the primary process of cation‐anion co‐intercalation. The superposition of blue (K_x_Nb_18_W_16_O_93_) and PG results in a deep blue appearance, characterized by low NIR transmittance and a slight reduction in VIS light transmittance (Figure 2C, yellow line, S3). The bright cooling state allows VIS light transmission while blocking most solar irradiance from NIR light (Figure 2F). As the negative bias increases to −2.4 V, PG further transforms into Prussian yellow (PY) due to enhanced Cl^−^ intercalation. The superposition of blue (K_x_Nb_18_W_16_O_93_) and yellow (PY) results in a deep‐colored ECD (Figure 2C, red line, S4), where transmittance of both VIS and NIR light is blocked. Once the bias is returned to OCP, the device restores to its initial state (Figure S2, Supporting Information). The corresponding chrominance coordinates L*, a*, and b* in the CIELAB color space are shown in Tables S1–S4 (Supporting Information). In summary, the distinct states of ECD demonstrate that its color and transmission spectral modulation can be controlled by cation (K^+^) and anion (Cl^−^) co‐intercalation/deintercalation. The varying transmittance spectra of PB, PW, PG, and PY highlight their potential for VIS light multi‐band modulation, while the coloring process of Nb_18_W_16_O_93_ exhibits dual‐band (VIS and NIR) electrochromism (Figure 2H‐J; Figures S3–S5, Supporting Information). The other electrochromic parameters—including response time, coloration efficiency, and cyclic stability—are presented in the Supplementary Information (Figures S6–S8, Supporting Information). These phenomena suggest supporting the ECD hierarchical regulation characteristics.

### 2.3 Hierarchical Energy‐Saving Control Strategy based on Building Load Demand

**Figure 3:**
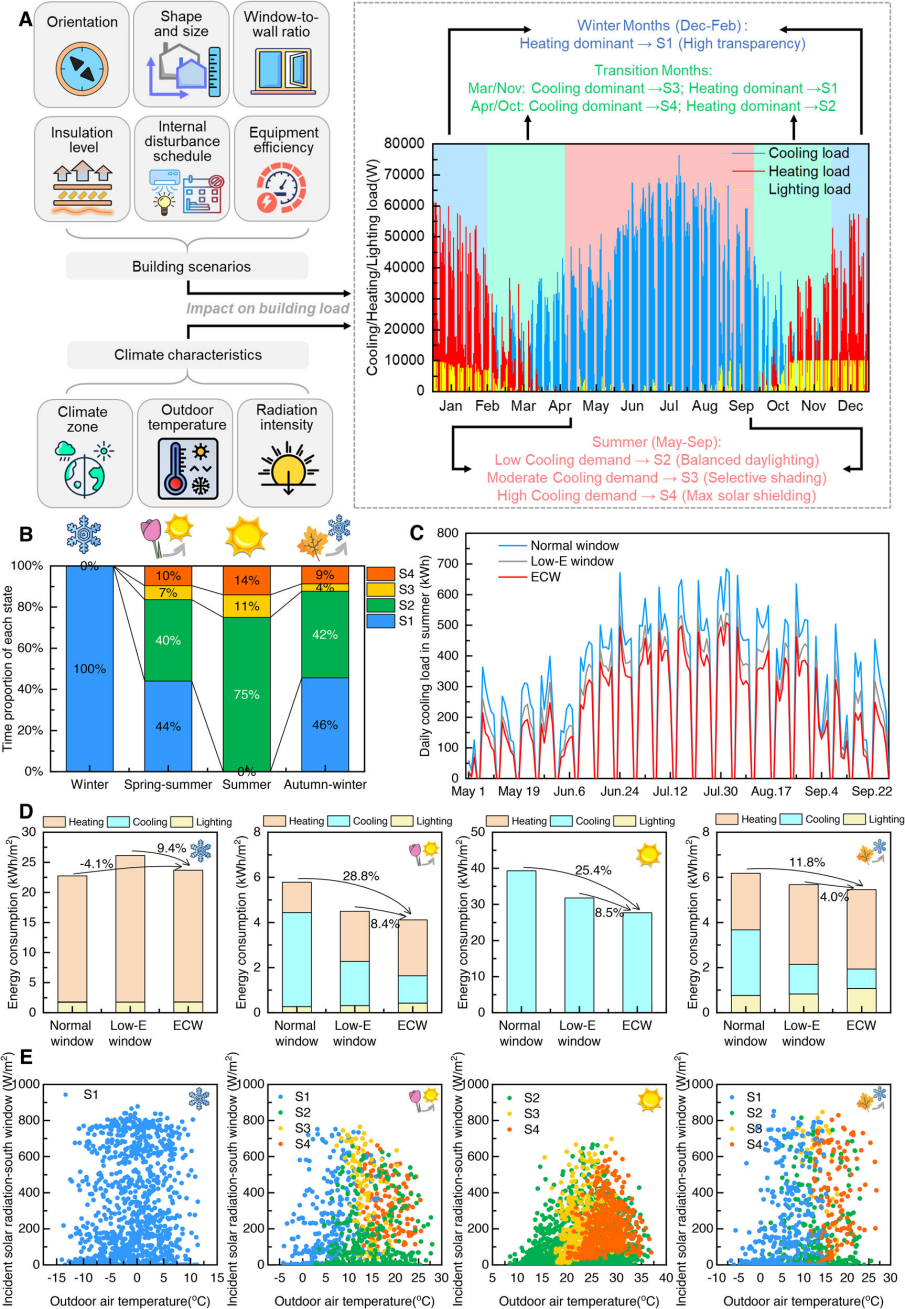
Hierarchical control strategy for electrochromic window (ECW) based on seasonal building demand. A) Load‐driven state transition logic. B) Seasonal state utilization profiles. C) Summer cooling load reduction compared to normal double‐layer insulating window. D) Annual energy performance across seasons. E) The coloring behavior and temperature‐radiation response characteristics of south‐facing electrochromic smart windows in different seasonal periods.

The current limitations of electrochromic technology lie in the insufficient consideration of practical control strategies in complex scenarios, leading to a disconnect between laboratory devices and building applications. We propose an innovative dynamic control framework centered on cooling and heating load demands as the core control logic, establishing a “seasonal mode‐dynamic hourly load‐spectral modulation capability” mapping regulation system (**Figure** **3**A).

First, Beijing was selected as a typical climate zone and a three‐story building model was established in EnergyPlus (Figures S9 and S10 and Table S5, Supporting Information). Based on the annual cooling or heating load distribution characteristics of the normal window baseline (Figure S11, Supporting Information), the operation cycle is divided into winter (December–February), summer (May–September), and the transitional seasons of spring‐summer (March,April) and autumn‐winter (October,November).

Leveraging the EnergyPlus Energy management system (EMS) module for continuous hourly thermal load monitoring,^.[^ ^52^ ^]^ the control strategy dynamically deploys the four optical states of the proposed ECW in response to real‐time load demand. This enables adaptive optical modulation where increasing load triggers prioritized reduction of near‐infrared transmission, followed by staged attenuation of visible light. Specifically, during summer operation, the control framework adopts a tiered state transition protocol based on real‐time cooling load demands, as shown in Figure 3A: low cooling load periods (<10 kW) activate the S2 state (T_vis_ = 38.4%, T_nir_ = 24.4%) to balance daylighting with initial heat gain mitigation; moderate cooling loads (10‐30 kW) trigger the S3 state (T_vis_ = 19.1%, T_nir_ = 11.2%) for selective near‐infrared radiation attenuation; while high cooling load conditions (>30 kW) engage the S4 state (T_vis_ = 10.5%, T_nir_ = 2.7%) to maximize solar heat gain rejection. The transitional seasons present more complex energy demand characteristics with alternating cooling and heating requirements, necessitating dynamic threshold strategies. Specifically, for March and November, the system employs S3 state during cooling‐dominant periods and reverts to S1 state when heating demands prevail; whereas April and October operations utilize S4 state for cooling‐dominated conditions and S2 state for heating‐dominant scenarios, ensuring optimal performance across varying seasonal transitions.

The effectiveness of this dynamic control strategy is clearly demonstrated through comprehensive state utilization analysis (Figure 3B; Figures S12–S17, Supporting Information). With a 1 h time step, Figures S12–S17 (Supporting Information) vividly depicts the sequential state transition characteristics and intuitively visualizes the optimal usage states for each time period. It clearly demonstrates that different states can be dynamically adjusted and applied according to seasons and time periods. Figure 3B quantifies the impact of climatic factors on load demand and the resulting modulation requirements for the transmittance of electrochromic windows by statistically analyzing the time proportions of states S1–S4 in different seasons and time periods. During summer cooling periods, the system optimally deploys states S2 (75% utilization), S3 (11%), and S4 (14%). This means that, completely energy‐free operation can be achieved most of the time through the brightly heated S2 state (open‐circuit voltage of 0), which can significantly reduce peak electricity demand during hot summers and alleviate pressure on the power grid. Transitional seasons exhibit more complex multi‐state dynamics, with utilization distributions of ≈45% (S1), 40% (S2), 5% (S3), and 10% (S4), confirming the strategy's ability to intelligently adapt to varying load conditions while minimizing unnecessary state transitions that could compromise performance.

The dynamic control strategy enables electrochromic smart windows to achieve superior daily cooling load reduction (Figure 3C; Figures S18–S20, Supporting Information). The cumulative daily energy savings translate into significant seasonal benefits (Figure 3D), with simulation results revealing a remarkable 25.4% reduction in summer cooling energy consumption versus normal windows and an 8.5% improvement over Low‐E windows, while maintaining lighting energy increases below 3%. This achievement represents a fundamental breakthrough in overcoming the inherent trade‐off between daylighting and thermal insulation that has traditionally limited electrochromic technology performance. The synergistic combination of spectrally tunable device properties and adaptive control algorithms lies at the heart of this advancement. The dynamic strategy proves equally effective during transitional seasons with alternating cooling and heating demands. For the spring‐summer transition period (March‐April), comprehensive energy savings reach 28.8% compared to normal windows and 8.4% versus Low‐E windows. Similarly, during the autumn‐winter transition (October‐November), the system maintains substantial energy savings of 11.8% and 4.0% compared to normal and Low‐E windows, respectively.

The energy‐saving performance of electrochromic smart windows is also governed by their orientation‐dependent interaction with spatiotemporal solar radiation patterns. Figure 3E and Figures S21–S23 (Supporting Information) elucidates distinct state transition pathways for electrochromic windows with different orientations. South‐facing windows (Figure 3E) in summer typically operate in S2 state (T_vis_ = 38.4%, T_nir_ = 24.4%) under low temperature and radiation conditions (<20 °C, <200 W/m^2^), progressively transitioning to S3 (T_vis_ = 19.1%, T_nir_ = 11.2%) and ultimately S4 (T_vis_ = 10.5%, T_nir_ = 2.7%) as solar intensity exceeds 300 W/m^2^, forming characteristic triangular state distribution patterns. In contrast, Figure S22 (Supporting Information) shows that west‐facing windows face more potential high radiation (>600 W/m^2^), corresponding to more intensive S4 state usage requirements, demonstrating the system's exceptional capability in managing challenging west‐facing solar heat gain scenarios. The universal applicability of this framework stems from its hierarchical integration of seasonal periodization, real‐time load tracking, and spectral state optimization, representing a paradigm shift from conventional material‐centric electrochromic window design to holistic built‐environment integration.

### 2.4 Hierarchical Regulation Strategy based on Climate Characteristics

**Figure 4:**
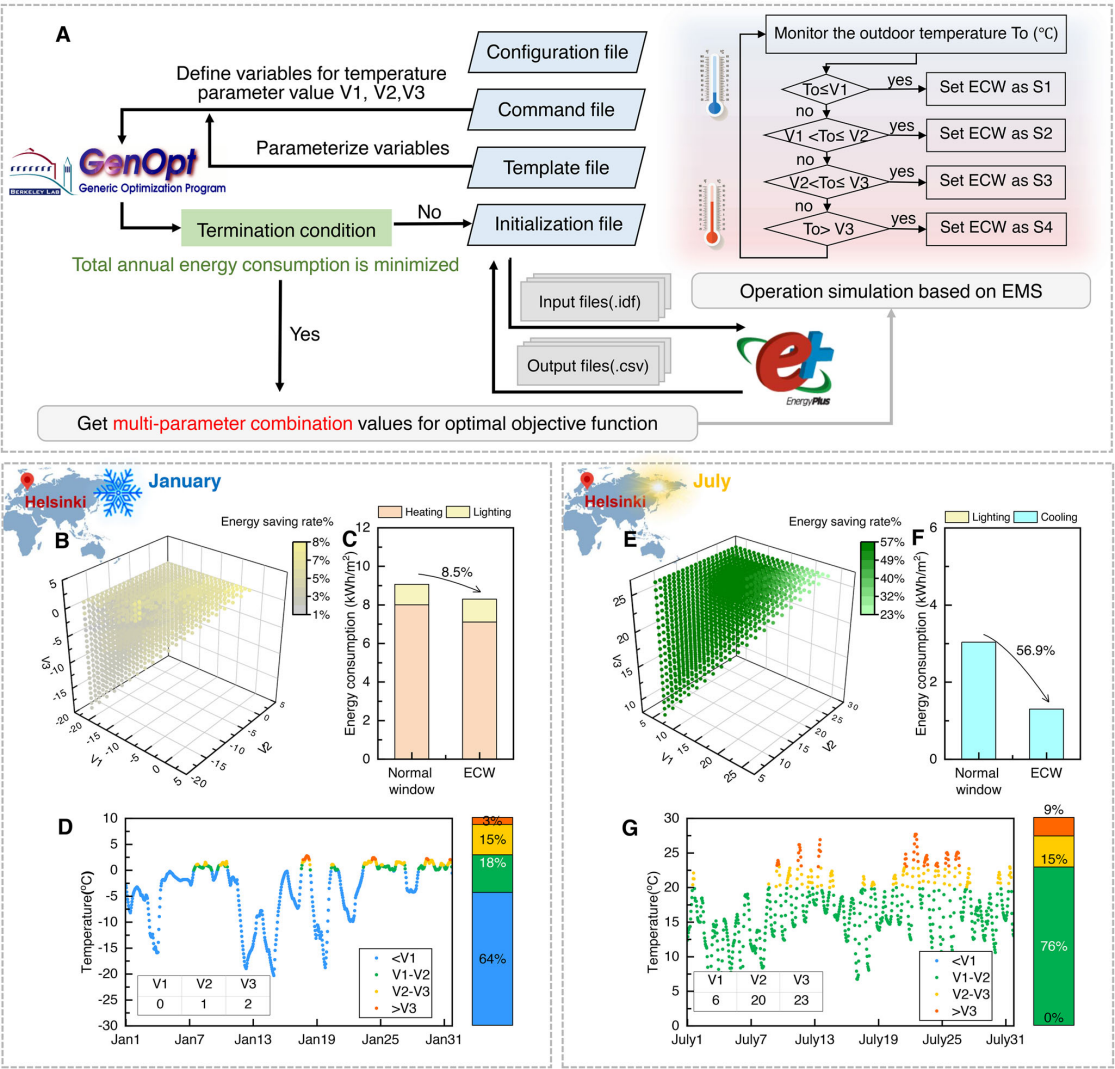
Hierarchical regulation strategy for electrochromic window (ECW) based on annual climate temperature characteristics. A) Temperature parameter optimization program and simulation process. B) All possible temperature control strategies and applications in Helsinki in January (each scattered point corresponds to a strategy, and the color is mapped to the energy‐saving rate relative to ordinary windows). C) The energy‐saving rate under the optimal strategy in Helsinki in January. D) Temperature‐state time series analysis under the optimal strategy in Helsinki in January. E) All possible temperature control strategies and applications in Helsinki in July. F) The energy‐saving rate under the optimal strategy in Helsinki in July. G) Temperature‐state time series analysis under the optimal strategy in Helsinki in July.

Beyond load‐driven control strategies, this study also proposes a universal temperature‐threshold‐driven framework (**Figure** **4**A), where three critical outdoor temperature value nodes (V1, V2, V3) autonomously regulate electrochromic windows across four optical states (S1–S4). Control strategies based on climate temperature thresholds are relatively simpler and more feasible, and climate parameters are decoupled from building configuration parameters, which can also significantly highlight the differences in the application of electrochromic technology across different climate zones.

A building energy model was established by EnergyPlus, encompassing three representative climate zones: hot (Abu Dhabi), temperate (Beijing), and cold (Helsinki). The climate characteristics are shown in Figure S24 (Supporting Information). Using a custom‐developed batch simulation platform, we performed exhaustive evaluations of energy‐saving rates for all feasible control strategies (V1‐V2‐V3 combinations) across January and July in these three climate zones. The threshold parameters were constrained within each city's historical temperature range (Table S6, Supporting Information). Based on the constraint of V1<V2<V3, a triangular distribution is generated in the 3D scatter plot, where each point represents a unique control strategy. Color mapping quantified energy‐saving rates relative to normal windows, with greener hues indicating higher efficiency. Scatter points in the lower‐left region corresponded to early‐state tinting at low temperatures, while upper‐right points reflected delayed activation under elevated thermal loads (Figure 4B,E; Figures S25A,D and S26A,D, Supporting Information).

An exhaustive evaluation of thousands of V1‐V2‐V3 combinations revealed striking disparities in annual energy consumption—up to 50% between strategies (Figure S26A, Supporting Information)—highlighting the critical role of control strategy parameter optimization in climate‐adaptive energy efficiency for electrochromic technology.

To identify globally optimal solutions, we developed a co‐simulation framework integrating EnergyPlus and GenOpt,^.[^ ^53^ ^]^ enabling joint optimization of threshold parameters across climates, followed by parameter invocation and performance simulation via EnergyPlus's EMS module.

Under the optimized control strategy, the electrochromic window significantly outperforms the static reference normal window. It achieves energy savings of 8.5% in January and 56.9% in July in Helsinki (Figure 4C,F), demonstrating its ability to overcome conventional limitations in cold regions.

Other cities have also shown encouraging applications (Figures S25 and S26, Supporting Information). Time‐resolved state‐switching analysis (Figure 4D,G; Figures S25 and S26, Supporting Information) demonstrated direct coupling between diurnal temperature fluctuations and window‐state transitions. This analysis meticulously depicts how the four typical states (S1–S4) dynamically shift to their optimal working states in real time, adapting to the changing outdoor temperatures. Threshold intervals rigorously aligned to regional climatic signatures. Specifically, Abu Dhabi's thresholds prioritized extreme heat mitigation, Beijing's balanced transitional‐season adaptability, and Helsinki's leveraged passive solar heating, revealing spatially tailored dynamic responses. This framework validated the flexibility of electrochromic windows in deploying state‐specific functionalities (S1–S4) at climatically optimal moments, redefining smart building envelope adaptability.

## 3 Conclusion

This work overcomes the fundamental disconnect between electrochromic material advances and building energy demands through a dual breakthrough. At the material level, we engineer a hierarchically regulated ECD assembled with Fe_4_[Fe(CN)_6_]_3_ and Nb_18_W_16_O_93_ film electrodes, achieving optical regulation via the cationic rocking‐chair and cation‐anion co‐intercalation mechanisms. This enables four typical spectral states: transparent heating (S1), bright heating for zero‐energy (S2), bright cooling for daylight‐preserving (S3), and dark cooling for maximum‐blocking (S4). Crucially, these material capabilities are transformed into real‐world impact via two pioneering control strategies: 1) A load‐responsive strategy focuses on internal building demands and adopts an “on‐demand adjustment” approach to address the adaptation of optical states, leveraging the self‐sustaining S2 state for >75% of summer operation to slash peak grid load while delivering 25.4% summer cooling savings versus normal double‐layer insulating windows—shattering the historic daylighting‐cooling compromise; 2) A universal climate‐adaptive strategy centers on external environmental constraints and employs a “location‐specific adjustment” method to resolve the presetting of operating thresholds based on regional climatic characteristics, revealing a staggering >50% performance gap between suboptimal and optimized controls. The framework achieves unprecedented performance across diverse climates, such as 56.9% energy savings in Helsinki during July. By integrating intelligent spectral control into building operations, we redefine electrochromic windows as adaptive building skins—dynamic regulators of energy flows across space and time. This shift from device‐centric to building‐aware design disrupts the performance degradation in smart window commercialization, offering a scalable solution for sustainable architecture. A dual breakthrough in hierarchical spectral modulation of material level and efficient building‐level control strategies maximizes energy savings of electrochromic windows—different strategies achieve over 50% performance variance. This paves a scalable path for translating laboratory innovations to real‐world building solutions, disrupting the traditional performance degradation chain in commercialization.

## 4 Experimental Section

### Materials

Ammonium niobate oxalate (C_4_H_4_NNbO_9_, 99.99%), ammonium paratungstate ((NH_4_)_10_H_2_(W_2_O_7_)_6_, >99%), oxalate dihydrate (C_2_H_2_O_4_·2H_2_O, ≥99%), anhydrous ethanol (C_2_H_5_OH, 99.8%), potassium ferricyanide (K_3_[Fe(CN)_6_], 99%), ferric chloride (FeCl_3_, 98%), acrylamide (AM, 99%), potassium persulfate (KPS, 99.5%), N, *N*‐methylenebisacrylamide (MBAA, 99%), potassium chloride (KCl, 99.5%). All chemicals were purchased from Shanghai Titan Scientific Co., Ltd (China), and used without further purification. Fluorine‐doped tin oxide (FTO) transparent conductive glasses (square resistance <15Ω sq^−1^, optical transmittance >83%) were purchased from Zhuhai Kaivo Optoelectronic Technology Co., Ltd (China).

### Preparation of Electrochromic Device—Growth of Nb_18_W_16_O_93_ films

Nb_18_W_16_O_93_ films were in situ grown on the surface of FTO coated glasses by hydrothermal method. First, the hydrothermal precursor solution can be obtained by following process. C_4_H_4_NNbO_9_ and (NH_4_)_10_H_2_(W_2_O_7_)_6_ were weighed according to an atomic ratio of niobium and tungsten as 9:8 and dissolved in 35 mL of 40% C_2_H_5_OH aqueous solution containing 5 g of C_2_H_2_O_4_⋅2H_2_O at room temperature. Second, the FTO glass should be diagonally inserted into a polytetrafluoroethylene (PTFE) liner containing 40 mL of precursor solution. After being kept at 180 °C for 4 h and cooled to room temperature, the films were taken out and cleaned with deionized water, and then dried at room temperature in air. The area of FTO glass is 2.5 × 5 cm^2^, and the area infiltrated by the hydrothermal precursor solution was 1.5 × 3 cm^2^.

### Preparation of Electrochromic Device—Growth of PB Films

For the electrodeposition of PB films, the electrolyte solution was composed of 0.05 mol L^−1^ of KCl, 0.01 mol L^−1^ of FeCl_3_ and 0.01 mol L^−1^ of K_3_[Fe(CN)_6_]. The electrodeposition was conducted under a constant current density of −50 µA cm^−2^ for 300 s. The area of FTO glass was 2.5 × 5 cm^2^, and the area infiltrated by the electrodeposition solution is 1.5 × 3 cm^2^.

### Preparation of Electrochromic Device—Assembly of the Device

The assembly of the device adopts a traditional sandwich structure. Two electrochromic electrodes were bonded with double‐sided tape, and then a gel electrolyte was constructed by *in‐situ* polymerization of PAM hydrogels. The preparation of PAM hydrogels is described below. First, 1.0 g of AM monomer and 0.7455 g of KCl were dissolved in 10.0 mL of deionized water. Then, 1 mg of MBAA cross‐linker and 5 mg of KPS initiator were added to the above AM solution. After stirring for 30 min, the solution was sonicated for 10 min to remove the dissolved air bubbles, and injected the gap of two electrodes separated by double‐sided tape. The cross‐linking polymerization were finished at 60 °C for 2 h to obtain PAM hydrogels.

### Preparation of Electrochromic Device—Measurements for Electrochemical and Electrochromic Properties

The electrochemical behavior of the Nb_18_W_16_O_93_ and PB films were investigated by using an electrochemical workstation (CHI 760E, China) in a three‐electrode system, where the FTO coated glasses grown Nb_18_W_16_O_93_ and PB films were used as the working electrodes, Pt electrode as the counter electrode, PAM hydrogel containing 1 mol L^−1^ of KCl as the electrolyte. Ag/AgCl electrode was used as the reference electrode. Electrochromic properties of the Nb_18_W_16_O_93_ and PB films were measured by using a fiber‐optical instrument from Ocean Optics (QEpro, Ocean Optics, USA) coupled to the electrochemical workstation. The effective optical range was from 380 to 2200 nm. The raw data of these were directly measured include the transmittance of the film and FTO glass, the raw data of the ECD was measured including the transmittance of the Nb_18_W_16_O_93_ film, PB film, PAM hydrogels and two layers of FTO glass.

### Building Simulation Details

1) *For Load Demand‐Based Control Strategy*: Building load demand was jointly determined by climatic characteristics and operational parameters. This study meticulously selects the climate of Beijing, a region with significant heating and cooling demands, to establish a benchmark model for a three‐story office building. Building configuration parameters, including envelope design, occupancy schedules, temperature setpoints, Heating, Ventilation and Air Conditioning (HVAC) system efficiency, and internal heat gains, were meticulously defined in accordance with established building energy efficiency standards. The east‐facing, south‐facing, and west‐facing facades feature a window‐to‐wall ratio of 70%. In the main text, a meticulous analysis was conducted to explore the disparities in the deployment of the four states of electrochromic window across different orientations. Seasonal and transitional control logic is implemented via the EnergyPlus Energy Management System (EMS) module. This module leverages its embedded scripting interface to enable real‐time tracking of building energy demand and dynamic switching of electrochromic window states.

These building configuration parameters fundamentally dictate electrochromic window state deployment strategies by directly influencing heat gain and thermal load profiles. The proposed load demand‐driven control framework demonstrates broad applicability. By adaptively mapping thermal load characteristics to optimize state selection, the framework can generate tailored, optimal control strategies for diverse heterogeneous scenarios.

2) *For Climate Temperature‐Based Control Strategy*: Building energy performance simulations were conducted utilizing the EnergyPlus EMS, which continuously monitors real‐time outdoor temperature data and dynamically adjusts the electrochromic window states at each simulation timestep (1 h). The state transition logic follows a temperature‐dependent hierarchy: the S1 state was activated when outdoor temperature falls below or equals threshold V1; the S2 state engages when temperature resides between V1 and V2; the S3 state initiates within the V2 to V3 interval; and the S4 state deploys when temperature exceeds V3. This framework enables adaptive modulation of glazing optical properties in direct response to climatic variations. Crucially, during state progression from S1 to S4, control prioritizes the reduction of near‐infrared (NIR) transmittance while sustaining relatively higher visible (VIS) transmittance within the photopic sensitivity range for human vision. This spectral prioritization strategy effectively mitigates solar heat gain while circumventing the substantial lighting energy penalties typically incurred by conventional devices due to excessive compromises in daylight harvesting efficiency.

Optimization of the critical temperature thresholds (V1, V2, V3) was achieved through an integrated co‐simulation framework coupling EnergyPlus with GenOpt, targeting annual total energy consumption (heating, cooling, lighting) minimization (Figure 4A). The workflow involved parameter initialization, objective function definition, and iterative optimization: GenOpt dynamically adjusted the values of V1, V2, and V3 over 2000 iterations, generating updated EnergyPlus input files and evaluating output files at each iteration to assess convergence criteria. This loop continued until termination conditions were met, yielding climate‐specific optimal parameters.

## Conflict of Interest

The authors declare no conflict of interest.

## Author Contributions

S.W. and J.S. contributed equally to this work. S.W. wrote the initial draft of the manuscript, including building application strategies, the Abstract, Conclusion, and Introduction. J.S. wrote the initial draft of the electrochromic device development and performance characterization sections and revised the manuscript. Z.D. contributed to the development of the electrochromic devices. X.L. supervised and participated in the electrochromic device development, providing technical guidance. J.X. developed the customized batch simulation platform and advised on simulation methodologies. H.S. and B.L. conceived the research concept, designed the framework, supervised the study, and revised the manuscript with critical feedback. All authors reviewed and approved the final manuscript.

## Supporting information



Supporting Information

## Data Availability

The data that support the findings of this study are available in the supplementary material of this article.
