# Supplementary material for: Building‐Tailored Hierarchical Electrochromism with Optimized Control Strategies
Source: Adv Sci (Weinh). 2025 Oct 16;12(47):e13530. doi: 10.1002/advs.202513530 (PMC12713013; doi:10.1002/advs.202513530)
Supplement: Supplementary file 1 — Supporting Information [file ADVS-12-e13530-s001.docx]

**Supplementary information for**

**Building-tailored Hierarchical electrochromism with Optimized Control Strategies**

**ShuangduiWu^2,3#^, Jiawei Sun^2,3#^, Zhoujie Duan^1^, Xiaolei Li^4^, Jun Xiao^2,3^, Hongli Sun^1,5*^, Borong Lin^2,3*^**

^1^College of Architecture and Environment, Sichuan University, Chengdu 610065, China

^2^Department of Building Science, Tsinghua University, Beijing 100084, China

^3^Key Laboratory of Eco Planning & Green Building, Ministry of Education, Tsinghua University, Beijing 100084, China

^4^Interdisciplinary Materials Research Center, Institute for Advanced Study, Chengdu University, China.

^5^State Key Laboratory of Hydraulics and Mountain River Engineering, Sichuan University, Chengdu 610065, China

*Corresponding author: E-mail: linbr@tsinghua.edu.cn, shl@scu.edu.cn

_

_

**Figure S1.** Transmittance spectra of electrochromic device (ECD) assembled with Fe_4_[Fe(CN)_6_]_3_ and Nb_18_W_16_O_93_ electrodes. Note: transparent heating, bright heating, bright cooling and dark cooling states are defined as S1, S2, S3 and S4, respectively.

**Table S1.** Chrominance coordinates L*, a*, b* at S1, S2, S3 and S4 of the ECD.

| State | L* | a* | b* |
| --- | --- | --- | --- |
| S1 | 87.26 | -0.83 | 7.3 |
| S2 | 76.58 | -19.29 | 0.99 |
| S3 | 65.11 | -31.35 | -8.7 |
| S4 | 44.13 | -11.05 | -5.57 |





**Figure S2.** Cyclic Voltammetry (CV) curve of the electrochromic device (ECD) assembled with Fe_4_[Fe(CN)_6_]_3_ and Nb_18_W_16_O_93_ electrodes—measured at a scan rate of 100 mV·s⁻¹ over the voltage range of -2.5 V to 1.6 V (blue line)—and the in-situ transmittance (at a wavelength of 633 nm, orange line). Note: transparent heating, bright heating, bright cooling and dark cooling states are defined as S1, S2, S3 and S4, respectively.





**Figure S3.** Cyclic Voltammetry (CV) curve of Fe_4_[Fe(CN)_6_]_3_ film electrode at a scan rate of 50 mV s^-1^ between −0.6 to 0.7 V.

The associated K^+^ ions intercalation process can be written as:

Fe_4_^Ⅲ^[Fe^Ⅱ^(CN)_6_]_3_ + 4K^+^ + 4e$\text{–}$**↔** K_4_Fe_4_^Ⅱ^[Fe^Ⅱ^(CN)_6_]_3_

(**PB**) (**PW**)

**Table S2.** Chrominance coordinates (L*, a*, b*) of Fe_4_[Fe(CN)_6_]_3_ film electrode at a scan rate of 50 mV s^-1^ between −0.6 to 0.7 V.

| State | L* | a* | b* |
| --- | --- | --- | --- |
| -0.6 | 99.28 | -4.91 | 17.37 |
| -0.4 | 98.88 | -5.5 | 17.04 |
| -0.2 | 98.17 | -6.6 | 16.62 |
| 0 | 95.6 | -11.52 | 16.74 |
| 0.2 | 91.51 | -16.79 | 17.4 |
| 0.4 | 90.08 | -19.36 | 17.82 |
| 0.6 | 90.79 | -18.07 | 19.29 |
| 0.7 | 90.26 | -18.85 | 18.36 |





**Figure S4.** Cyclic Voltammetry (CV) curve of Nb_18_W_16_O_93_ electrode at a scan rate of 50 mV s^-1^ between -1.8 to 0.2 V.

The intercalation of K^+^ ions and can be written as:

Nb_18_W_16_O_93_ + *x*K^+^ + *x*e$\text{–}$**↔** K*_x_*Nb_18_W_16_O_93_

**(transparent) (colored)**

**Table S3.** Chrominance coordinates (L*, a*, b*) of Nb_18_W_16_O_93_ electrode at a scan rate of 50 mV s^-1^ between -1.8 to 0.2 V.

| State | L* | a* | b* |
| --- | --- | --- | --- |
| -1.8 | 40.8 | -6.86 | -33.28 |
| -1.6 | 54.34 | -10.11 | -27.12 |
| -1.4 | 66.64 | -9.92 | -18.84 |
| -1.2 | 80.39 | -5.92 | -7.75 |
| -1.0 | 87.57 | -2.49 | -1.59 |
| -0.8 | 90.97 | -0.88 | 1.15 |
| -0.6 | 92.45 | -0.66 | 1.82 |
| -0.4 | 92.57 | -0.65 | 1.7 |
| -0.2 | 92.6 | -0.65 | 1.65 |
| 0 | 92.62 | -0.65 | 1.67 |
| 0.2 | 92.59 | -0.64 | 1.73 |





**Figure S5.** Cyclic Voltammetry (CV) curve of Fe_4_[Fe(CN)_6_]_3_ film electrode at a scan rate of 50 mV s^-1^ between 0.6 to 1.4 V.

The associated intercalation of Cl^−^ ions can be written as:

Fe_4_^Ⅲ^[Fe^Ⅱ^(CN)_6_]_3_ + *x*Cl$\text{–}$ **↔** Fe_4_^Ⅲ^[Fe^Ⅲ^(CN)_6_]*_x_*Cl*_x_*[Fe^Ⅱ^(CN)_6_]_3-_*_x_* + *x*e$\text{–}$, 0*<x*<3

(**PB**) (**PG**)

Fe_4_^Ⅲ^[Fe^Ⅲ^(CN)_6_]*_x_*Cl*_x_*[Fe^Ⅱ^(CN)_6_]_3-_*_x_* + (3-*x*) Cl$\text{–}$ **↔** Fe_4_^Ⅲ^[Fe^Ⅲ^(CN)_6_]_3_Cl_3_ + (3-*x*) e$\text{–}$

(**PG**) (**PY**)

**Table S4.** Chrominance coordinates (L*, a*, b*) of Fe_4_[Fe(CN)_6_]_3_ film electrode at a scan rate of 50 mV s^-1^ between 0.6 to 1.4 V.

| State | L* | a* | b* |
| --- | --- | --- | --- |
| 0.6 | 64.76 | -13.38 | -31.69 |
| 0.8 | 79.92 | -15.4 | -10.6 |
| 1 | 85.16 | -11.82 | 2.81 |
| 1.2 | 90.57 | -3.39 | 15.86 |
| 1.4 | 93.26 | -0.06 | 19.33 |





**Figure S6.** *In-situ* transmittance curve (at a wavelength of 633 nm) of the electrochromic device (ECD) assembled with Fe_4_[Fe(CN)_6_]_3_ and Nb_18_W_16_O_93_ electrodes, measured over a voltage range of -2.4 V (20 s) to 1.5 V (20 s).





**Figure S7.** Changes in the optical density at 633 nm with respect to the intercalated charge density.





**Figure S8.** Cycling performance of the device under the square-wave (−2.4 V for 15 s and 1.3 V for 15 s) cycling for 4000 cycles.


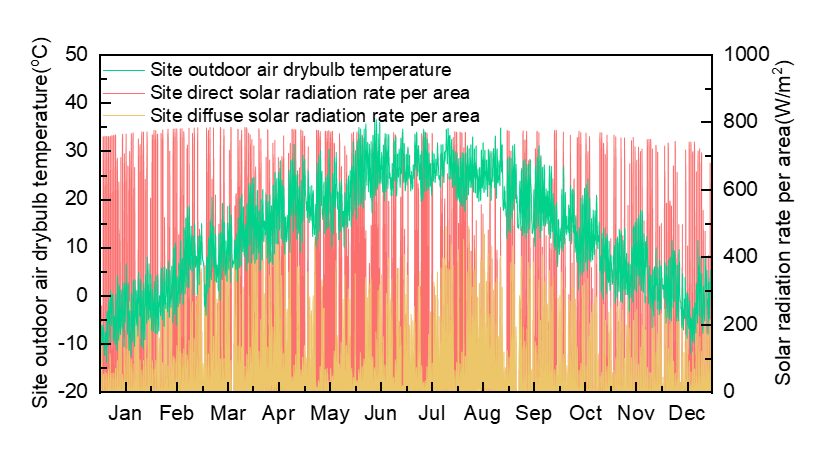


Figure S9. The climate characteristics of Beijing.


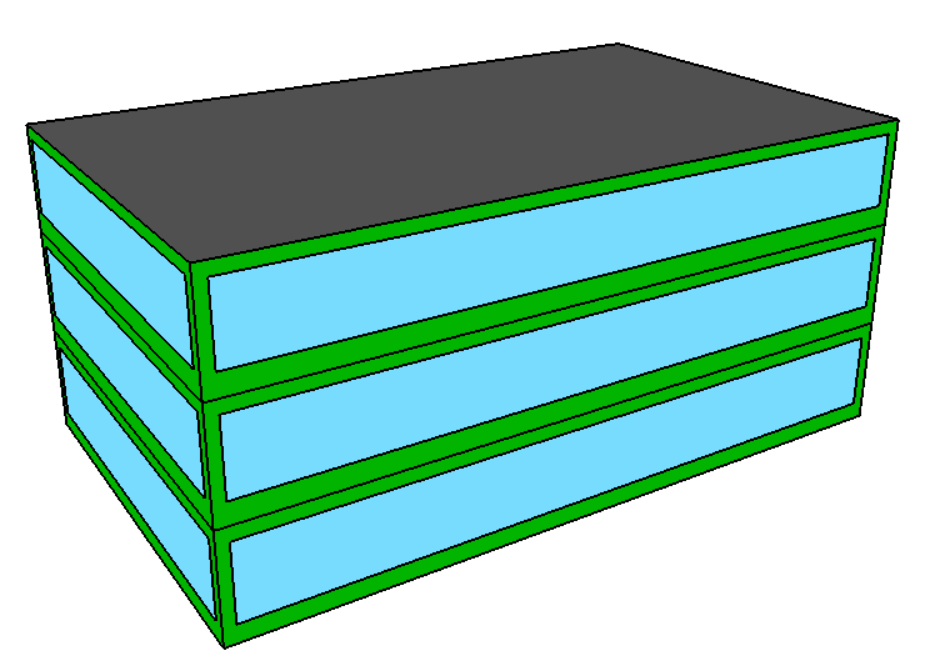


**Figure S10.** Building model for dynamic load-driven control strategy (20*12*3 each layer).

Table S5. Building model and related parameter setting for load-driven simulation.

| Parameter | Setting |
| --- | --- |
| Envelope | The heat transfer coefficient of the external envelope is 0.4 W/m^2^·K  70%WWR in south、east、west orientation |
| Indoor load | occupancy density: 10 m^2^ /person  lighting power density: 9 W/ m^2^ |
| Working schedule | 8:00 to 18:00 form Monday to Friday |
| Design parameter | The heating and air conditioning temperatures in the room were set to 20°C and 24°C |
| Lighting control | Linear/off strategy, target illuminance at 500lx |
| Equipment for HVAC | Heat pumps are used for heating and cooling, both with coefficient of performance (COP) 2.0 |

Figure S11. The annual load variation curve based on the benchmark normal window.

Figure S12. Temporal characteristics of electrochromic window states under load-driven optimization strategy during summer periods. Remarkably, the predominant utilization of the S2 state (green-shaded regions in the figure) achieves zero power consumption due to the unique self-bleaching properties of Prussian blue-based electrochromism. The inherently low power consumption of the devices, coupled with high-efficiency control algorithms, facilitates practical low-energy operation of electrochromic smart windows.

Figure S13. Temporal characteristics of electrochromic window states under load-driven optimization strategy during spring-summer transition periods.

Figure S14. Temporal characteristics of electrochromic window states under load-driven optimization strategy during autumn-winter transition periods.

Figure S15. State transition timeline of electrochromic windows on a typical summer day (Aug. 19).

Figure S16. State transition timeline of electrochromic windows on a typical autumn-winter Transition-day (Oct. 25).

Figure S17. State transition timeline of electrochromic windows on a typical autumn-winter Transition-day (Nov. 29).

Figure S18. The reduction of the daily cooling load of electrochromic smart windows during the spring-summer transition season under the load-driven optimization strategy.

Figure S19. The reduction of the daily cooling load of electrochromic smart windows during the autumn-winter transition season under the load-driven optimization strategy.

**Figure S20.** The reduction of the daily cooling load of electrochromic smart windows in the winter season under the load-driven optimization strategy.


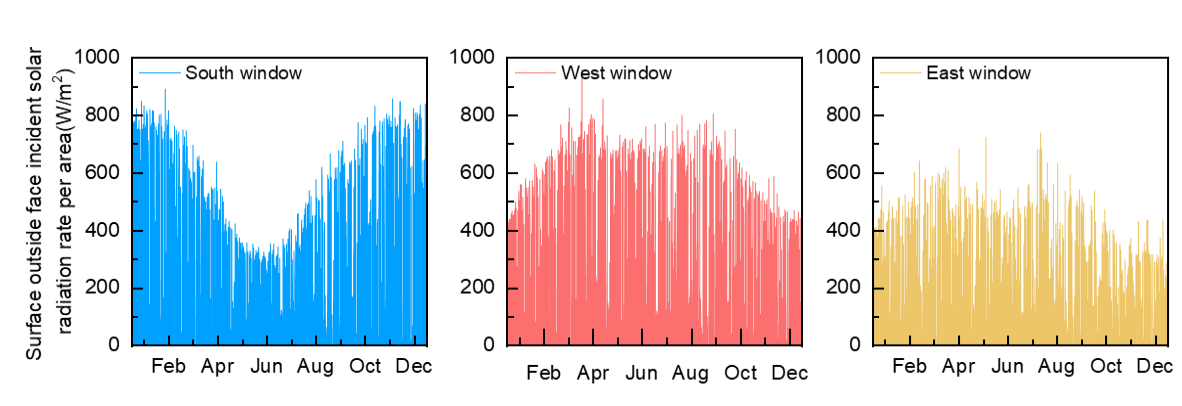


Figure S21. The differences in incident solar radiation on the surfaces of east-west and south-facing windows.


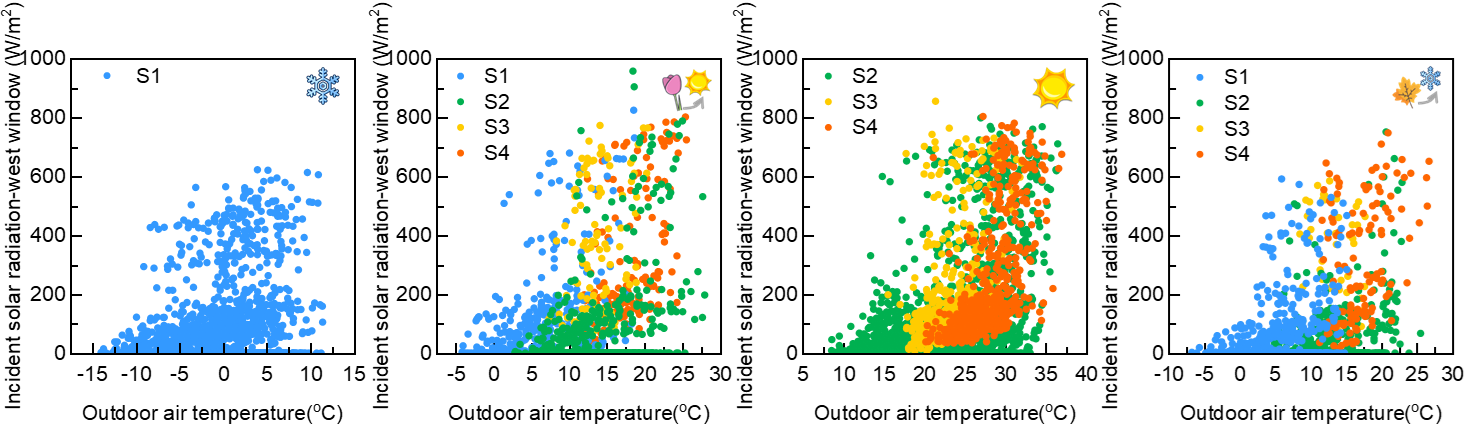


Figure S22. The coloring behavior and temperature-radiation response characteristics of west-facing electrochromic smart windows in different seasonal periods.


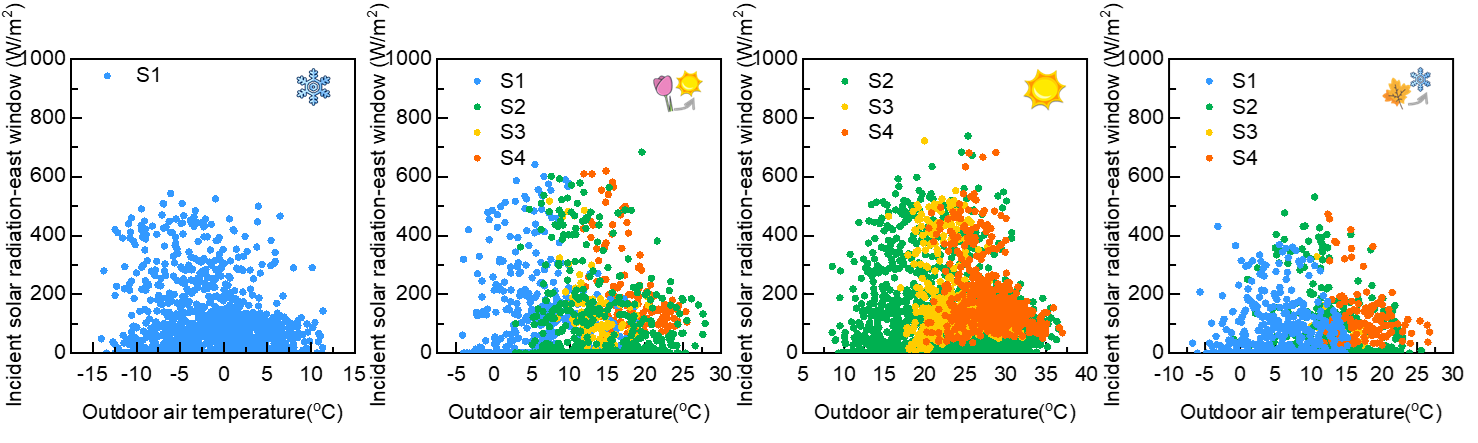


**Figure S23.** The coloring behavior and temperature-radiation response characteristics of east-facing electrochromic smart windows in different seasonal periods.


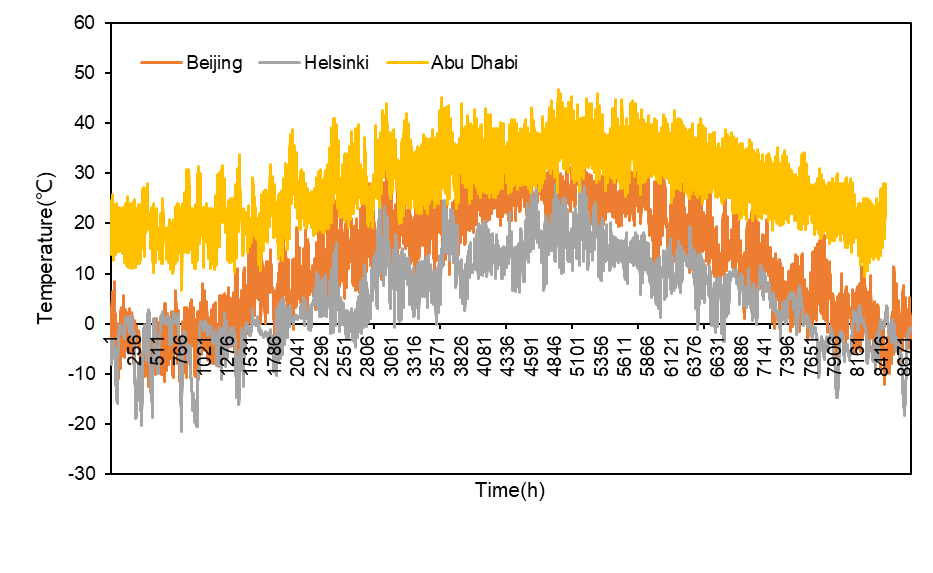


Figure S24. The climate characteristics used in climate-based control strategy.

Table S6. The temperature ranges in Beijing, Helsinki and Abu Dhabi during typical winter and summer months.

| Temperature | Beijing | Helsinki | Abu Dhabi |
| --- | --- | --- | --- |
| The maximum temperature in January | 8 | 3 | 28 |
| The minimum temperature in January | -14 | -20 | 10 |
| The maximum temperature in July | 35 | 28 | 47 |
| The minimum temperature in July | 19 | 6 | 26 |


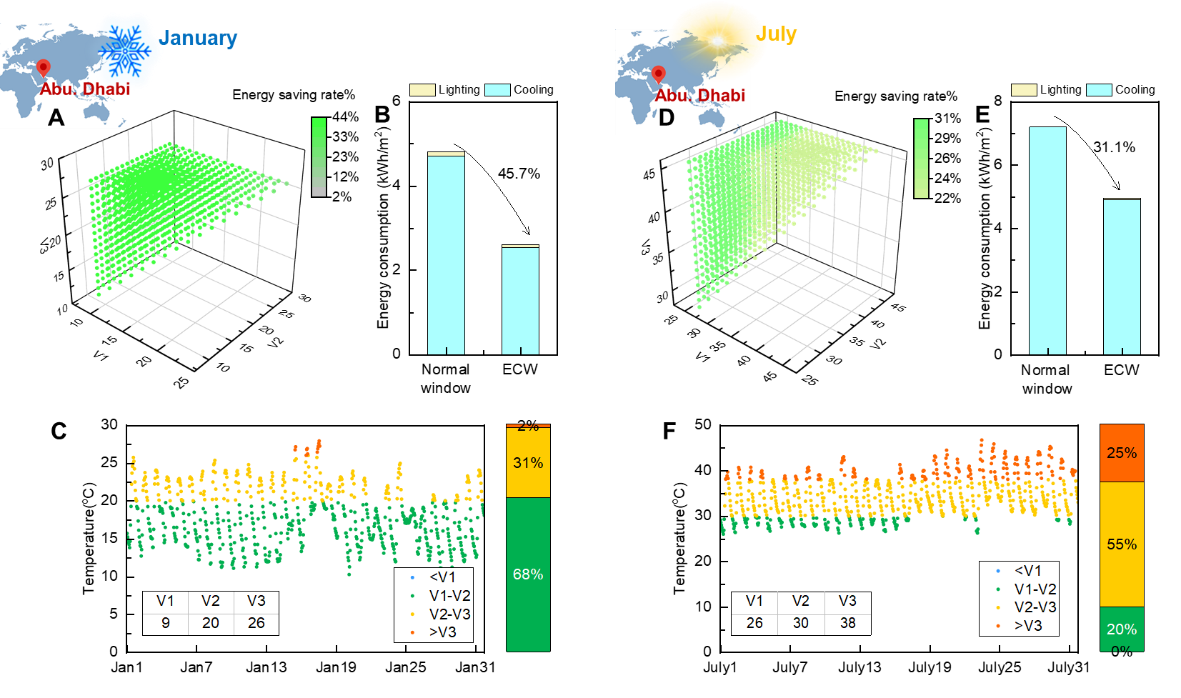


Figure S25. Climate-driven adaptive control strategy and application of electrochromic windows

(A) All possible temperature control strategies and applications in Abu Dhabi in January.

(B) The energy-saving rate under the optimal strategy in Abu Dhabi in January.

(C) Temperature-state time series analysis under the optimal strategy in Abu Dhabi in January.

(D) All possible temperature control strategies and applications in Abu Dhabi in July.

(E) The energy-saving rate under the optimal strategy in Abu Dhabi in July.

(F) Temperature-state time series analysis under the optimal strategy in Abu Dhabi in July.


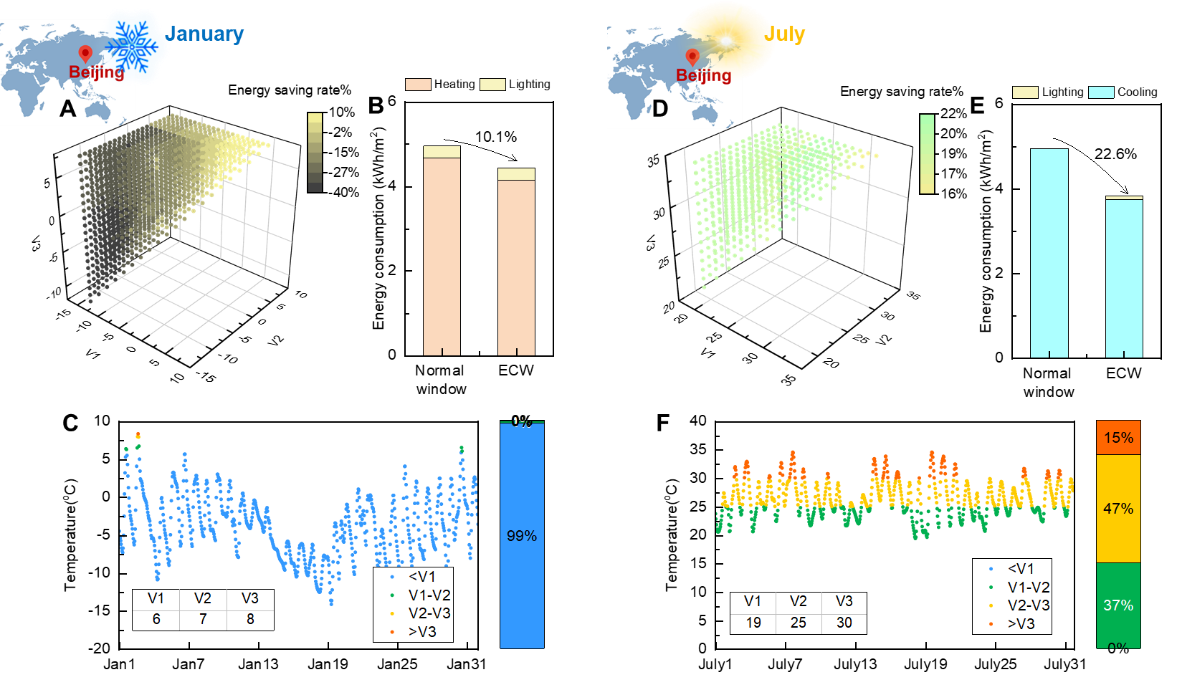


Figure S26. Climate-driven adaptive control strategy and application of electrochromic windows

(A) All possible temperature control strategies and applications in Beijing in January.

(B) The energy-saving rate under the optimal strategy in Beijing in January.

(C) Temperature-state time series analysis under the optimal strategy in Beijing in January.

(D) All possible temperature control strategies and applications in Beijing in July.

(E) The energy-saving rate under the optimal strategy in Beijing in July.

(F) Temperature-state time series analysis under the optimal strategy in Beijing in July.
